# Recurrent laryngeal nerve monitoring in esophagectomy is easy to use and feasible in both open and minimally invasive surgery

**DOI:** 10.1016/j.xjtc.2025.02.005

**Published:** 2025-02-21

**Authors:** Adam Zeyara, Knut Olanders, Oscar Åkesson

**Affiliations:** a Department of Clinical Sciences, Faculty of Medicine, Lund University, Lund, Sweden; b Upper GI Surgery Division, Department of Surgery, Skåne University Hospital, Lund, Sweden; c Department of Anesthesiology and Intensive Care, Skåne University Hospital, Lund, Sweden

**Figure:**
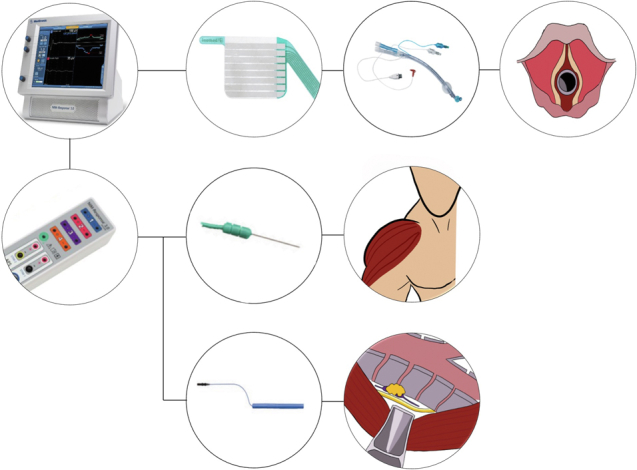
A schematic view of our NIM setup.

**Central Message**

We conclude that recurrent laryngeal nerve monitoring in esophagectomy is easy to use and feasible in both open and minimally invasive surgery. In select cases it might be an important aid to reduce recurrent laryngeal nerve injuries.

Recurrent laryngeal nerve palsy is a considerable contributor to morbidity and mortality after esophagectomy.^1^ The incidence of recurrent laryngeal nerve (RLN) palsy after esophagectomy depends mainly on tumor site, extent of lymphadenectomy, and surgical approach. Incidence can also differ significantly between centers as the result of different definitions and follow-up programs. In international literature, the incidence of RLN palsy after esophagectomy ranges from 9% to 22%,^2^, ^3^, ^4^ but some Asian centers have reported incidences of up to 59%,^5^ probably as the result of extensive routine lymphadenectomy. The surgical challenges in avoiding RLN injury largely depend on its close relations with the superior mediastinal lymph nodes and the difficulties in obtaining optimal visualization in this area. Although existing data suggest that superior mediastinal lymph nodes, such as position 106, are common sites of metastases,^6^, ^7^, ^8^, ^9^, ^10^, ^11^, ^12^, ^13^, ^14^ many centers choose to compromise the potential long-term benefits of removing these nodes routinely to avoid the harming effects of RLN palsy.^15^^,^^16^

Skåne University Hospital in Lund is a Swedish tertiary and national reference center for esophageal and gastric surgery, performing approximately 45 transthoracic (2- and 3-field) esophagectomies yearly. Ivor Lewis esophagectomies are usually performed with a hybrid technique (laparoscopy for the abdominal part and thoracotomy for the thoracic part), whereas most McKeown esophagectomies are performed with totally minimally invasive technique (thoracoscopy, laparoscopy, and left cervical incision). Validated data from 500 of our esophagectomies showed a postoperative RLN palsy incidence of 9.3%. We mainly perform Ivor Lewis esophagectomies and do not routinely harvest the superior mediastinal lymph nodes, nevertheless, RLN palsy seems to be a significant problem in our practice. Therefore, we decided to start a pilot project for using nerve integrity monitoring (NIM) when planning for an Ivor Lewis procedure with an anastomosis above the azygos vein and for all McKeown esophagectomies.

NIM is a technique that enables surgeons to identify and monitor nerve function in real time. There are numerous different variants and set-ups, but the 2 main types are continuous and intermittent monitoring. During continuous monitoring, the surgeon is presented with a continuous real-time curve while during intermittent monitoring the surgeon must manually stimulate the nerve with a handheld probe to get a signal. The continuous type is more cumbersome to install and requires some additional surgical dissection.

NIM has been used for decades in thyroid and ear-nose-throat (ENT) surgery with very good results.^17^ During the last years, the use of NIM in esophagectomy has increased,^18^^,^^19^ especially during minimally invasive surgery, and in particular when harvesting high-risk superior mediastinal lymph nodes such as position 106. Multiple systematic reviews suggest that NIM may reduce the incidence of RLN palsy after esophagectomy.^18^^,^^19^ Nevertheless, NIM is still a novelty to most esophageal centers, and specific technical set-ups will be needed for different centers.

Many centers have described their experiences from implementing NIM in cervical and transthoracic esophageal procedures before. Most of them involve a single-lumen endotracheal tube with a preattached laryngeal electrode and a bronchial blocker for one-lung ventilation.^20^ Double-lumen endotracheal tubes with a preattached laryngeal electrode are not available. In our clinic, anesthesiologists prefer using a double-lumen endotracheal tube for one-lung ventilation. Only 3 technical descriptions were found in PubMed-indexed journals using a double-lumen endotracheal tube. One of them was 13 year old and described only 1 case.^21^ The other described a series of 6 patients.^22^ The most recent one, published by the Mainz team in 2020, is a very thorough description of the technique in 10 robot-assisted esophagectomy cases.^23^ The technique of using NIM with a double-lumen endotracheal tubes with manually attached laryngeal electrodes in esophagectomy is not new; however, evidence is limited. This Surgical Technique article should primarily be seen as our initial experience of this technique and a practical aid for esophageal centers that are interested in implementing NIM. However, apart from describing practical details, the main research questions were to assess the detection rate and specificity of the method, as well as investigating whether anesthesia/preoperative time and operating time were significantly affected.

## Methods

A retrospective observational cohort study was performed of all patients undergoing esophagectomy with NIM from January 1, 2022, to December 31, 2022, as part of our pilot project.

### Data Collection and Calculations

The variables surgical approach, *International Classification of Diseases* code (histology), level of esophagogastric anastomosis (centimeters from incisor teeth measured by endoscopy), RLN signal after dissection, anesthesia/preoperative time and operating time, postoperative voice status, and ENT follow up were extracted.

Detection rate and specificity were calculated. Anesthesia and preoperative prep times and operating time were compared with esophagectomies performed during the previous year (2021) for significant differences. All data are available upon request.

### Outcomes

The primary end points were detection rate and specificity. Secondary end points were differences in anesthesia and preoperative time and operating time.

### Statistical Analyses

All analyses were conducted using the IBM SPSS Statistics software for Mac, version 29.0.1.0 (Build 171).

### Ethics

The study was approved by the local research ethics committee of Stockholm (Dnr: 2013-59631 and 2020-06495).

### Practical Details

The nerve integrity monitor system from Medtronic, NIM 3.0 (used by endocrine surgeon colleagues in our department) was modified to be applicable to our procedures.

Before designing the setup, we agreed on a number of terms, namely that the system:

• be compatible with the entire panorama of our esophagectomy approaches (open/hybrid/totally minimally invasive);
• be easy to set up (no additional surgical dissection);
• not significantly increase the time for preoperative preparations;
• be easy to use and interpret; and
• be compatible with a double-lumen endotracheal tube, which is the preferred choice for one-lung ventilation by our anesthesiologists (over single-lumen tube with bronchial blocker).

A summarized version of our setup is as follows:

1. A double-lumen endotracheal tube is prepared with an adhesive laryngeal electrode (Figure 1).

**Figure 1:**
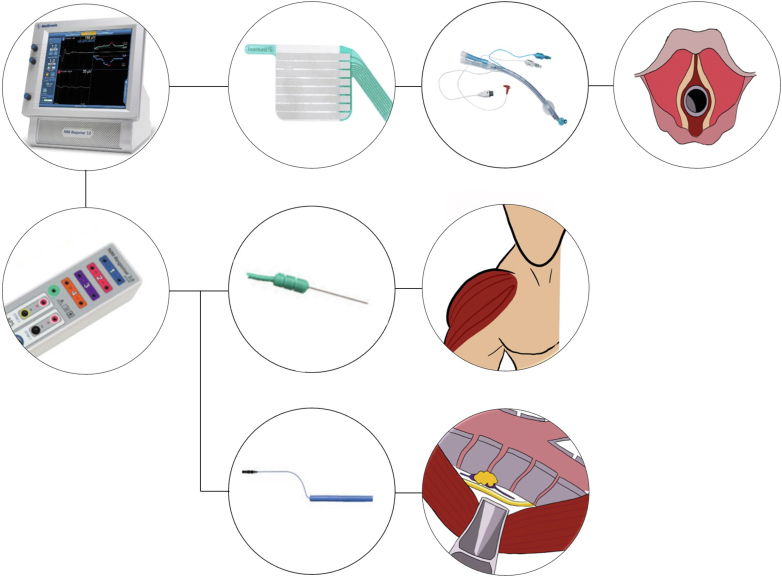
A schematic view of our NIM setup. *First row:* The NIM 3.0-console is connected to the laryngeal electrode (Inomed Medizintechnik GmbH), which in turn is attached to a Ch 37 or 39 double-lumen tube (Ambu VivaSight or RÜSCH Bronchopart). The tube is then inserted with the electrode at the level of the vocal cords. *Second row:* The NIM 3.0-controller (which is connected to the console) is connected to the ground electrodes which are placed on the right deltoid muscle. *Third row:* The NIM 3.0-controller is connected to the monopolar handheld stimulator probe (Medtronic), which is used to stimulate the nerve. Photos of the NIM 3.0-console, NIM 3.0-controller, laryngeal electrode, double-lumen tube, ground electrodes, and handheld stimulator are subjected to Copyright by Medtronic, Ionomed and Ambu. The drawings of the laryngeal inlet, human silhouette with highlighted deltoid muscle and the surgical view of the position 106 lymph node and left recurrent laryngeal nerve are subjected to copyright by the author Adam Zeyara.
2. The correct position of the laryngeal electrode is confirmed during intubation.
3. The ground electrodes are placed on the right deltoid muscle (Figure 1).
4. The thyroid program is used on the NIM-response console and the stimulator is set on 2.0 mA only recognizing events over 100 μV (Figure 1).
5. The intraoperative detection is performed using a handheld stimulator probe, which is elongated with a stiff suction irrigation probe for thoracoscopic cases (Figure 2).

**Figure 2:**
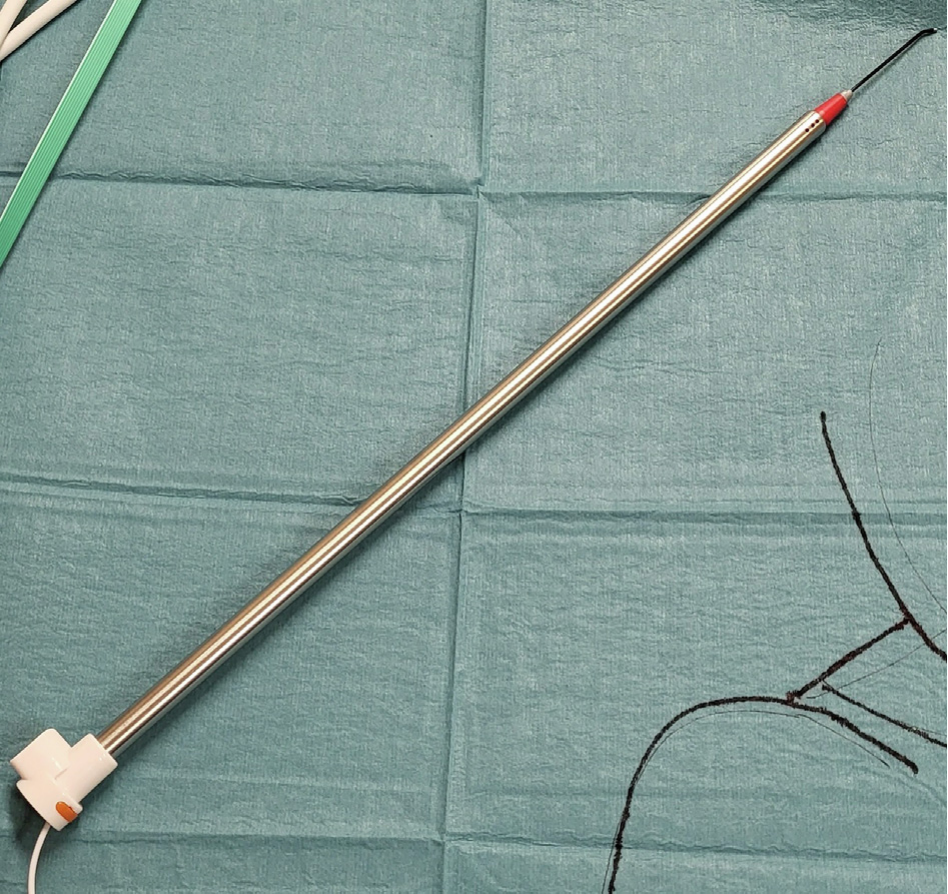
The handheld stimulator probe was elongated using a stiff suction irrigation probe (Apgar A/S) for thoracoscopic cases.
6. Without neuromuscular blockade, the operating field may be substantially impaired by vigorous diaphragmatic breathing which must be actively suppressed by opioids such as remifentanil.

### Detailed Walkthrough

#### Anesthesia

Before induction of general anesthesia, an epidural catheter was inserted at the Th7-10 level. General anesthesia was induced by propofol, fentanyl, and rocuronium and the patient was intubated using a video laryngoscope (Ambu Glidecope or Storz C-mac; Macintosh blade) with a left-sided double-lumen tube (Ambu VivaSight or RÜSCH Bronchopart) size Ch 37 or 39. Before intubation, the double-lumen tube was prepared with an adhesive Inomed Laryngeal Electrode Select (Inomed Medizintechnik GmbH) as described to follow (Figure 1). The correct position of the laryngeal electrode being at the level of the vocal cords was confirmed during intubation. Anesthesia was maintained with sevoflurane, fentanyl, and rocuronium during the abdominal stage and the epidural was activated before the thoracotomy. Neuromuscular blockade was reversed with neostigmine-glycopyrrolate or sugammadex during the thoracic stage, making intraoperative nerve monitoring (IONM) possible. During neuromuscular blockade, the operating field may be substantially impaired by vigorous diaphragmatic breathing which must be actively suppressed by opioids such as remifentanil.

##### Attachment of the adhesive laryngeal electrode on a double-lumen tube

Failure of IONM of the RLN is common because of malposition of the endotracheal tube, leading to lack of connection between the electrodes and the vocal cords. Using a single-lumen tube, as well as adjustments in depth and rotation, often corrects this problem.

For one-lung ventilation, a double-lumen tube was used, which is the preferred way of separate the lung ventilation in our practice, although a single-lumen tube and a bronchial blocker could have been an alternative. However, a double-lumen tube cannot be adjusted in depth and rotation, to correct a lack of signal from the IONM system, as it is possible with a single-lumen tube. The placement of the adhesive laryngeal electrodes on the double-lumen tube is thus critical.

We used the adhesive Inomed Laryngeal Electrode Select (Inomed Medizintechnik GmbH) intended for single lumen tubes with 7- to 9-mm inner diameter. For separated lung ventilation, we used left-sided double-lumen tubes (AmbuVivaSight or RÜSCHBronchopart) size Ch 37 or 39.

It was crucial that the adhesive electrodes were placed at a correct distance from the distal cuff to an estimated level of the vocal cords, enabling both one-lung ventilation and RLN monitoring. We measured the distance from the carina to the vocal cords on a preoperative computed tomography scan and added 1 to 1.5 cm on the basis of experiences of previous cases. This distance was then measured from the proximal limit on the distal bronchial cuff to a location proximal on the tube, where a mark was made. The adhesive electrode was then positioned central over the marker, leaving a small gap on the anterior side, as the result of the larger double-lumen tube. This is now changed, leaving the gap on the posterior side for potentially even better contact with the vocal cords.

Intubation was performed using a video laryngoscope assuring that the electrodes were positioned correctly at the level of the vocal cords and a bronchoscope was used to control the distal end of the double lumen tube. When a VivaSight tube was used, bronchoscopy was often not necessary. The length of the electrode field at the level of the vocal cords is 3.5 cm, enabling for minor miscalculations and yet a good contact and a functioning RLN monitoring. A correct position and good contact with the vocal cords was also confirmed with the four check marks on the Medtronic monitor.

#### Preoperative and operative aspects

The ground electrodes were placed percutaneously on the right deltoid muscle and attached to the Medtronic NIM 3.0 system (Figure 1). Intraoperative detection was performed using a monopolar hand-held stimulator probe (Medtronic), which was elongated using a stiff suction irrigation probe (Apgar A/S) for thoracoscopic cases (Figure 2). We used the Thyroid program on the NIM-response console (Medtronic) and the stimulator was set on 2.0 mA only recognizing events over 100 μV (Figure 1).

**Figure 3:**
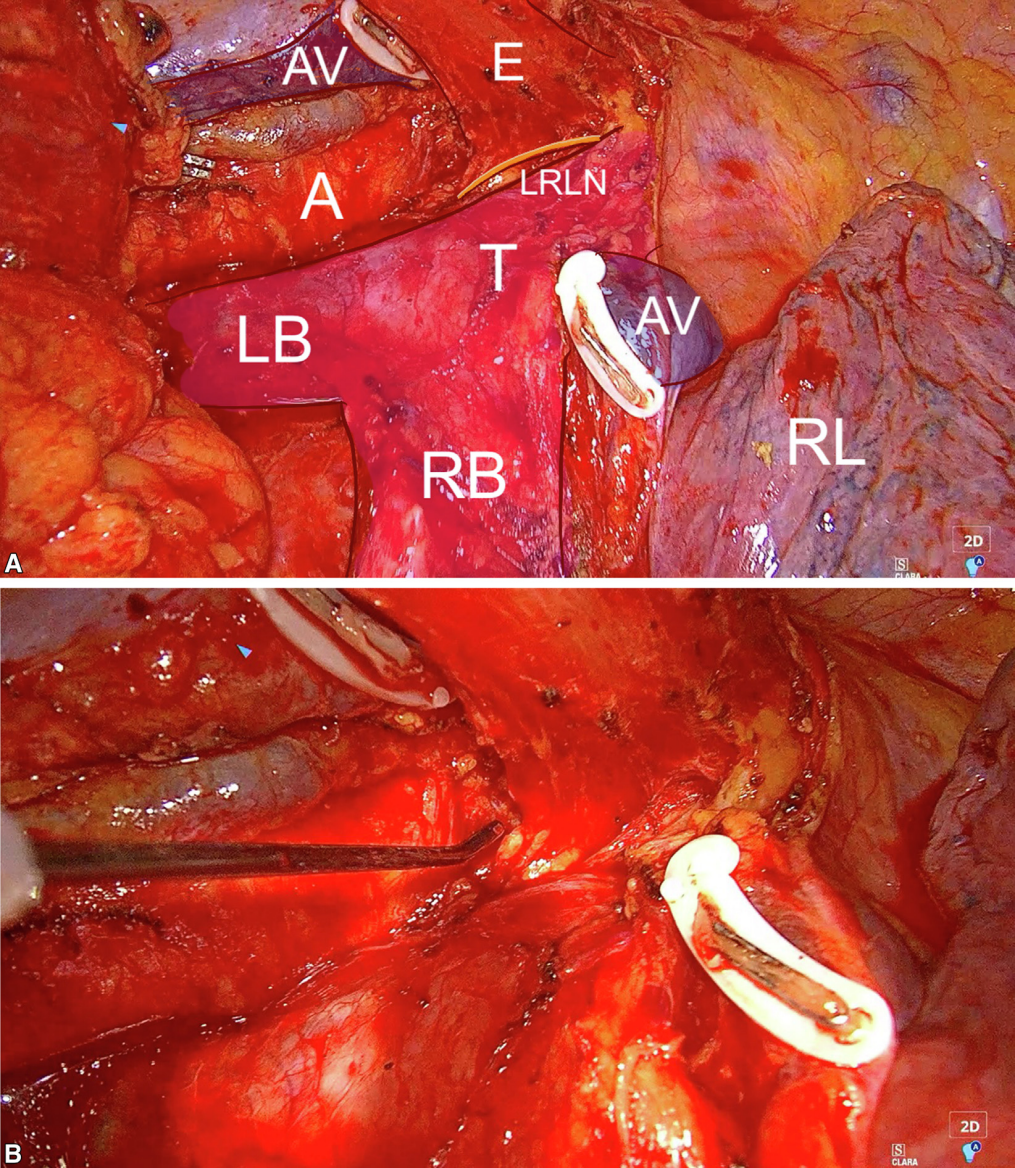
A, Still image from a live recording of a right-sided thoracotomy view of an Ivor Lewis esophagectomy. The right lung (*RL*) is collapsed and retracted, and the azygos vein (*AV*) has been divided. The esophagus (*E*) has been dissected from the aorta (*A*) and the left and right bronchi (*LB*, *RB*) as well as the trachea (*T*). The cephalad traction of the proximal esophagus causes a slight left upwards tilt of the trachea and the left paratracheal space in which the left recurrent laryngeal nerve (*LRLN*) runs, exposing it for potential injury or transection. B, Still image from live recording of a right-sided thoracotomy view of an Ivor Lewis esophagectomy. The NIM probe is used to map the course of the left recurrent laryngeal nerve. The course of the nerve can be mapped out long before it is visible to the naked eye, allowing for safe dissection despite interindividual anatomic variations or difficult surgical conditions as the result of locally advanced disease or previous high doses of radiation therapy, which is often the case in squamous cell carcinoma.

Figure 3, *A*, is a still image from a live recording of a right-sided thoracotomy view of an Ivor Lewis esophagectomy. When operating in the superior mediastinum, the cephalad traction of the proximal esophagus causes a slight left upwards tilt of the trachea and the left paratracheal space in which the left RLN runs, exposing it for potential injury or transection (Figure 3, *A*). Figure 3, *B* and Video 1 show how the course of the nerve can be mapped out long before it is visible to the naked eye, allowing for safe dissection despite, for example, interindividual anatomic variations or difficult surgical conditions attributable to locally advanced disease or previous high doses of radiation therapy, which is often the case in squamous cell carcinoma.

## Results

In total, 14 patients who underwent esophagectomies (10 Ivor Lewis and four McKeown) during 2022 had esophagectomy with NIM (Table 1). All anastomoses were either cervical or at the level of the superior thoracic aperture. Intraoperative nerve identification was successful in 11 cases. In 1 case, only the right RLN could be identified, probably as the result of injury. In 2 cases, nerve identification failed altogether. Of the 2 cases, one was caused by tumor overgrowth (the procedure was stopped) and the other was probably attributable to inadequate muscle relaxant reversal. Of the 11 cases in which both nerves were identified, 10 had clinically normal voice immediately postoperatively and one was hoarse but eventually regained full vocal cord function as assessed by an ENT-specialist after 3 months.

**Table 1:** Cases included in this series and selected data

Case	Surgical approach	Tumor ICD code (histology)	Anastomosis (centimeters from incisor teeth)	Intact RLN signal after dissection	Clinical postoperative voice status	Postoperative ENT follow-up
1	Ivor Lewis	C160B, Siewert II (ADC)	23 cm (superior thoracic aperture)	Yes	Hoarse	Normal 3 mo postop
2	McKeown	C154, middle esophagus (SCC)	20 cm (cervical)	Yes	Normal	−
3	Ivor Lewis	C160B, Siewert II (ADC)	Thoracic (superior thoracic aperture)	Yes	Normal	−
4	McKeown (salvage)	C153, upper esophagus) (SCC)	18 cm (cervical)	Yes	Unknown∗	−
5	Ivor Lewis	C154, middle esophagus (SCC)	25 cm (superior thoracic aperture)	Yes for right RLN, no for left RLN	Hoarse	Left RLN palsy 6 mo postop
6	Ivor Lewis	C160A, Siewert I (ADC)	23 cm (superior thoracic aperture)	Yes	Unknown†	
7	McKeown	C154, middle esophageal (SCC)	−	No	Patient was hoarse already preoperatively‡	−
8	Ivor Lewis	C154, middle esophageal (SCC)	24 cm (superior thoracic aperture)	Yes	Normal	−
9	Ivor Lewis	C160A, Siewert I (ADC)	Thoracic (superior thoracic aperture)	Yes	Normal	−
10	McKeown	C160A, Siewert I (ADC) + long segment Barrett's	Cervical	Yes	Normal	−
11	Ivor Lewis	C160A, Siewert I (ADC)	25 cm (superior thoracic aperture)	No	Normal	−
12	Ivor Lewis	C160A, Siewert I (ADC)	25 cm (superior thoracic aperture)	Yes	Normal	−
13	Ivor Lewis	C160A, Siewert II (ADC)	23 cm (superior thoracic aperture)	Yes	Normal	Normal 6 mo postop
14	Ivor Lewis	Long segment Barrett's with high-grade dysplasia	24 cm (superior thoracic aperture)	Yes	Normal	−

*ICD*, *International Classification of Diseases*; *RLN*, recurrent laryngeal nerve; *ENT*, ear-nose-throat; *ADC*, adenocarcinoma; *SCC*, squamous cell carcinoma.

∗ Patient was never extubated due to conduit necrosis, eventually deceased.

† Patient was never extubated because of multiple postoperative complications and eventually died.

‡ Patient was scheduled for an open McKeown but the procedure was stopped because of tumor overgrowth on trachea.

Both RLN s were identified in 11 of 14 cases. We chose to consider the case in which only the right nerve was identified as a failure because the left nerve was not identified before injury. In all cases in which the nerve signal was intact after dissection, patients also had normal voice or eventually a normal ENT status. Detection rate and specificity were our primary end points, detection rate was 78% and specificity was 100%.

Mean anesthesia and preoperative prep time with NIM was 96.0 ± 30.8 minutes and mean operating time with NIM was 368.8 ± 66.2 minutes. These were compared by means of an independent *t* test with the mean anesthesia and preoperative time (89.3 ± 15.2 minutes) and mean operating time (335.1 ± 45.4 minutes) from 30 random esophagectomies without NIM from the previous year. The mean differences were 7.9 minutes longer anesthesia and preoperative prep times in the NIM-group and 33.7 minutes longer operating time in the NIM-group. The differences were not statistically significant, *P* = .055 and *P* = .207, respectively. See Table 2 for a summary.

**Table 2:** Anesthesia and preoperative prep times and operating time with and without NIM

Outcome measure	Esophagectomy with NIM, min, mean ± SD	Esophagectomy without NIM, min, mean ± SD	Mean difference, min	*P* value∗
Anesthesia and preoperative prep times	96.0 ± 30.8	89.3 ± 15.2	7.9	.055
Operating time	368.8 ± 66.2	335.1 ± 45.4	33.7	.207

*NIM*, Nerve integrity monitoring; *SD*, standard deviation.

∗ *P* > .050 was not considered significant.

## Discussion

This Surgical Technique article describes our implementation of intraoperative NIM during the esophagectomy procedure. With no previous training, we developed a reliable and reproducible setup universally applicable to all our surgical approaches. It could easily be used with a double-lumen endotracheal tube as preferred by our anesthesiologists. After a short learning curve, the system was easy to install. There were no significant failures with regards to the electrode attachment on the endotracheal tube. There were no other significant hardware or software malfunctions. There were no adverse experiences from operating without muscle relaxants on the neck or in the thorax. Surgically, as expected, it was of particular aid during dissection and lymphadenectomy above the level of the azygos vein. We have decided to continue the use of this technique for select cases.

In summary, intraoperative mapping has taught us that the anatomical threat of especially the left RLN during dissection above the level of the azygos vein is imminent, regardless of whether the superior mediastinal nodes are harvested. We believe that many of our RLN injuries probably occur during this part of the dissection. In our hands, NIM was easy to install, detection rate and specificity were high, and anesthesia and preoperative prep time as well as operating time were not significantly prolonged. We conclude that NIM for the esophagectomy procedure is possible without previous training and is a safe and possibly important aid to reduce RLN injuries. However, much larger samples in a randomized setting would be needed to assess its true effectiveness in this context.

## Conflict of Interest Statement

The authors reported no conflicts of interest.

The *Journal* policy requires editors and reviewers to disclose conflicts of interest and to decline handling or reviewing manuscripts for which they may have a conflict of interest. The editors and reviewers of this article have no conflicts of interest.

## Supplementary Data

Video 1 Video available at: https://www.jtcvs.org/article/S2666-2507(25)00072-0/fulltext.
