# Supplementary figures and images for: Recurrent laryngeal nerve monitoring in esophagectomy is easy to use and feasible in both open and minimally invasive surgery
Source: JTCVS Tech. 2025 Feb 21;31:188–94. doi: 10.1016/j.xjtc.2025.02.005 (PMC12237746; doi:10.1016/j.xjtc.2025.02.005)

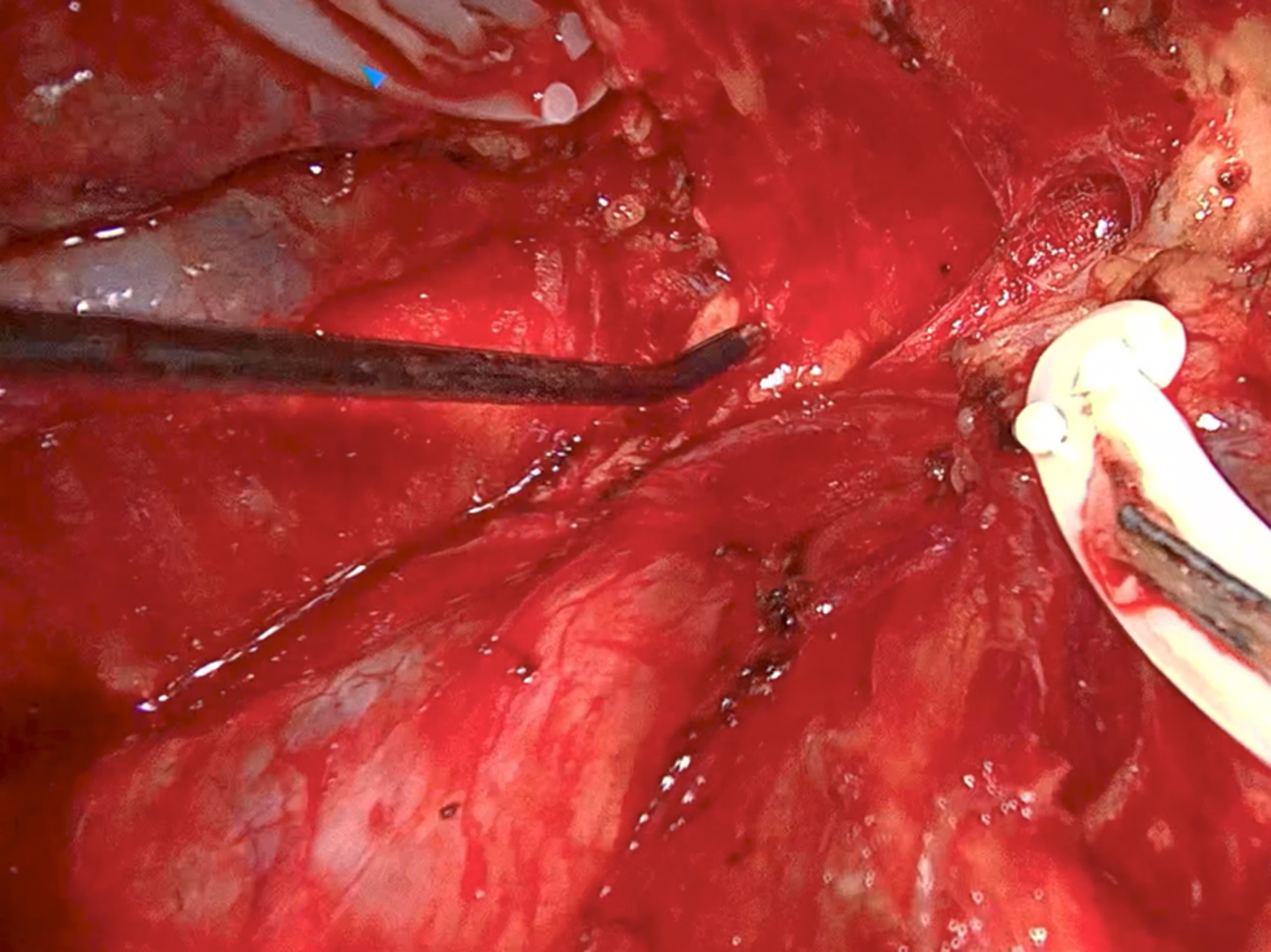

Supplement: Video 1 — Video available at: https://www.jtcvs.org/article/S2666-2507(25)00072-0/fulltext. [file fx2.jpg]
